# Comparative phylogeography of Atlantic reef fishes indicates both origin and accumulation of diversity in the Caribbean

**DOI:** 10.1186/1471-2148-8-157

**Published:** 2008-05-22

**Authors:** Luiz A Rocha, Claudia R Rocha, D Ross Robertson, Brian W Bowen

**Affiliations:** 1 Hawaii Institute of Marine Biology, University of Hawaii, P.O. 1346, Kaneohe, HI 96744, USA; 2 Smithsonian Tropical Research Institute, Balboa, Republic of Panamá

## Abstract

**Background:**

Two processes may contribute to the formation of global centers of biodiversity: elevated local speciation rates (the center of origin hypothesis), and greater accumulation of species formed elsewhere (the center of accumulation hypothesis). The relative importance of these processes has long intrigued marine biogeographers but rarely has been tested.

**Results:**

To examine how origin and accumulation affected the Greater Caribbean center of diversity, we conducted a range-wide survey of mtDNA cytochrome *b *in the widespread Atlantic reef damselfish *Chromis multilineata *(N = 183) that included 10 locations in all four tropical Atlantic biogeographic provinces: the Greater Caribbean, Brazil, the mid-Atlantic ridge, and the tropical eastern Atlantic. We analyzed this data and re-evaluated published genetic data from other reef fish taxa (wrasses and parrotfishes) to resolve the origin and dispersal of mtDNA lineages. Parsimony networks, mismatch distributions and phylogenetic analyses identify the Caribbean population of *C. multilineata *as the oldest, consistent with the center of origin model for the circum-Atlantic radiation of this species. However, some Caribbean haplotypes in this species were derived from Brazilian lineages, indicating that mtDNA diversity has not only originated but also accumulated in the Greater Caribbean. Data from the wrasses and parrotfishes indicate an origin in the Greater Caribbean in one case, Caribbean origin plus accumulation in another, and accumulation in the remaining two.

**Conclusion:**

Our analyses indicate that the Greater Caribbean marine biodiversity hotspot did not arise through the action of a single mode of evolutionary change. Reef fish distributions at the boundaries between Caribbean and Brazilian provinces (the SE Caribbean and NE Brazil, respectively) indicate that the microevolutionary patterns we detected in *C. multilineata *and other reef fishes translate into macroevolutionary processes and that origin and accumulation have acted in concert to form the Greater Caribbean biodiversity hotspot.

## Background

The extremely high biodiversity at the two global coral reef hotspots – the Indo-Malay Archipelago (IMA, also known as the Coral Triangle) in the Indo-Pacific, and the Greater Caribbean (GC) region in the Atlantic – has long intrigued marine biologists [1-8]. Two primary hypotheses have been proposed to explain such richness and the corresponding biodiversity gradients moving away from those regions: the center of origin (CO) hypothesis, introduced by Darwin as "centers of creation" [9], proposes that species originate in the center and disperse to the periphery, and the high central diversity arises through *in-situ *speciation [10]. According to Briggs, the most prominent contemporary supporter of this hypothesis, centers of diversity "act as centers of evolutionary radiation and supply species to other areas that are less effective in an evolutionary sense" [10-12]. Evidence from recent phylogenetic [13] and species-distribution surveys [10,14] support the CO model for the IMA. In contrast, the center of accumulation (CA) hypothesis proposes that diversity centers accumulate species that originated elsewhere. The IMA lies on the western boundary of the Pacific and westward flowing ocean currents could transport the pelagic larvae of species originating anywhere in the Pacific to the IMA [15,16]. A recent analysis of reef fish and coral distributions in the Indian and Pacific Oceans concluded that deviations from a random species-richness pattern predicted by a mid-domain model are consistent with this hypothesis [17].

Genetic surveys of sea urchins, marine gastropods and cowries in the Indo-Pacific indicate that species formation has occurred throughout the region [4,18,19], both inside the center of diversity (supporting the CO hypothesis) and outside the IMA (supporting the CA hypothesis). Likewise, the reef fish genera *Thalassoma *and *Halichoeres *display no clear pattern, as ancient and recent species occur both in the IMA and elsewhere in the Pacific [20,21]. These and other studies have led to the proposition that both origin and accumulation of species contribute to the high diversity of the IMA [5,6,19,21].

Patterns of genetic variation within widely distributed species can offer clues that may indicate how origin and accumulation contribute to a center of diversity [22]. There are four ways in which intra-specific genetic variation can contribute pertinent information: (i) Resolution of the geographic locations of both phylogenetically ancestral or basal DNA sequences (haplotypes) and recent or derived haplotypes. Under a CO scenario, basal lineages should be found at the center of diversity. In contrast, the restriction of ancestral haplotypes to peripheral populations would support the CA model. (ii) Patterns of variation in genetic diversity throughout the species range could also be informative. Under the CO hypothesis, higher haplotype and nucleotide diversities should occur in the diversity centers, but away from the center under the CA hypothesis. Note, however, that the value of such evidence is limited because at equilibrium, the largest population will have the highest diversity, regardless of age. Further, (iii) mismatch distributions and population genetic analyses may offer other useful clues about the geography of origination and subsequent dispersal: in old, widely distributed species, basal haplotypes (which assume an interior position in parsimony networks) and derived haplotypes (peripherally located in parsimony networks) may occur in all populations. However, if the species recently expanded its range the younger populations would be less variable, may exhibit star-like parsimony networks with a few very common haplotypes and many rare haplotypes, and would have a Poisson-like mismatch distribution [23-25]. Finally, (iv) direction of migration can also be informative: gene flow away from the center to the periphery would support the CO hypothesis whereas the reverse flow would favor CA [26].

**Figure 1:**
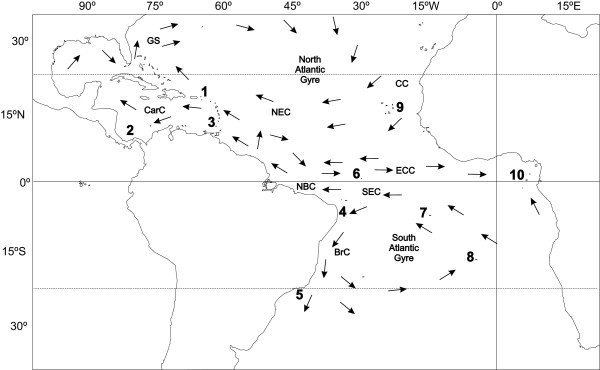
**Sampling locations and Ocean currents**. Sampling locations and major tropical Atlantic Ocean currents. Locations are: 1. St. Croix, USVI; 2. San Blas, Panama; 3. Grenada; 4. Paraiba; 5. Cabo Frio; 6. St. Paul's Rocks; 7. Ascension; 8. St. Helena; 9. Cape Verde; 10. Sao Tome. Current abbreviations are: GS, Gulf Stream; CarC, Caribbean Current; NEC, North Equatorial Current; NBC, North Brazil Current; CC, Canary Current; SEC, South Equatorial Current; ECC, Equatorial Counter Current; BrC, Brazil Current.

While attention has been focused on evolutionary mechanisms producing the Indo-Pacific center of diversity, the CO and CA hypotheses have never been tested in the tropical Atlantic Ocean. The tropical Atlantic is an appropriate forum for such a test because geographically and oceanographically it is a much simpler system than the western Pacific, and, due to its isolation from the Indo-Pacific, allows independent tests of the proposed mechanisms of diversity production. This area comprises four tropical biogeographic provinces: the Greater Caribbean (the Caribbean itself, the Antilles, the Gulf of Mexico, Florida, the Bahamas and Bermuda); Brazil (the coastline and oceanic islands south of the Equator to 28°S); the mid-Atlantic ridge (Ascension and St. Helena islands), and the tropical eastern Atlantic (from Cape Verde to Angola, including Cape Verde and the Islands in the Gulf of Guinea) [27,28] (Fig. 1). Geologically the mid-Atlantic ridge includes St. Paul's rocks (off northeastern Brazil) as well as Ascension and St. Helena. However, effects of geographic proximity mandate that the shore fishes of St. Paul's are more closely related to the Brazilian fauna [29].

The biogeographic barriers separating these provinces include vast geographic and oceanic distances lacking suitable habitat: the northeastern South American coast is heavily influenced by freshwater outflow, and there is no coral reef development in the 2,300 km wide area between the Amazon's mouth and Trinidad & Tobago; the eastern and western Atlantic, as well as the central Atlantic islands, are separated from the other provinces by thousands of kilometers of deep open ocean [28,30]. Previous mtDNA surveys of reef fishes and sea urchins have revealed deep phylogenetic breaks among these four tropical biogeographic provinces [20,31,32], as well as the existence of some species that can apparently transcend some of the barriers through dispersal of pelagic larval and juvenile stages [33-38].

Here we analyze patterns of genetic diversity based on mtDNA sequences in a common, widespread Atlantic reef fish – the brown chromis (*Chromis multilineata*) across all four tropical Atlantic biogeographic provinces. The combination of this transatlantic distribution and the lack of a marked genetic break between populations in the two western Atlantic provinces (see Results) makes it a good candidate to study the roles of origin versus accumulation in explaining high species diversity in the Greater Caribbean. Additionally, we re-evaluated data from four species groups, published in two previous studies of Atlantic reef fishes [36,38] that reported genetic lineages shared by Brazil and the Caribbean. Our main objectives were: 1) to search for signatures of origin vs. accumulation of genetic diversity in the Greater Caribbean, which is the Atlantic center of diversity for tropical reef organisms; and 2) to assess how the barriers between major biogeographic provinces influence the population structure of *C. multilineata*, a widely distributed reef fish with a relatively short pelagic larval stage.

## Results

An 802 bp segment from the cytochrome *b *gene was analyzed for 183 individuals obtained from ten locations (Fig. 1) spanning the entire geographical range of *Chromis multilineata *on both sides of the tropical Atlantic. A total of 121 polymorphic sites distributed among 132 haplotypes were identified for those individuals. Mean nucleotide frequencies were A = 0.24, C = 0.35, G = 0.15, T = 0.26. The transition – transversion ratio was 8.8:1. Average pairwise distances between populations ranged from 0.01 to 1.8 mean nucleotide substitutions (*d *= 0.001% to 0.22% sequence divergence) within the western/central Atlantic, and from 7.1 to 7.9 mean nucleotide substitutions (*d *= 0.88% to 0.98% sequence divergence) between the eastern Atlantic and the other three provinces. Haplotype diversity (*h*) in *C. multilineata *was very high, with unique haplotypes present in 71.8% of the individuals. Populations in Caribbean, in Brazil and in the central Atlantic each had slightly higher haplotype diversity (*h *= 0.92 – 1.0), than the tropical eastern Atlantic populations (*h *= 0.81 – 0.92), although those differences are not statistically significant. Nucleotide diversity (π) was low in all populations, but highest (although not significantly higher) in the Caribbean (Table 1).

**Table 1:** *Chromis multilineata *populations data

	N	H	*h*	π	*PD*	Fu's *Fs*	*R2*	τ	θ_0_	θ_1_
*Greater Caribbean*										
Grenada	16	14	0.97 ± 0.03	0.009 ± 0.005	7.125	-5.631* (0.006)	0.0864* (0.026)	5.94	2.61	65.68
St. Croix (USVI)	25	23	0.99 ± 0.01	0.008 ± 0.004	6.203	-17.536* (0.000)	0.0475* (0.000)	6.84	0.10	122.97
Panama	19	19	1.00 ± 0.02	0.008 ± 0.005	6.602	-15.011* (0.000)	0.0632* (0.001)	4.33	2.70	3839.37
*Central Atlantic Islands*										
Ascension	19	16	0.98 ± 0.02	0.007 ± 0.004	5.356	-8.637* (0.001)	0.0797* (0.024)	6.18	0.00	77.65
St. Helena	20	19	0.99 ± 0.02	0.007 ± 0.004	5.126	-15.492* (0.000)	0.0585* (0.001)	5.80	0.00	4001.25
*Brazil*										
St. Paul's Rocks	17	10	0.92 ± 0.04	0.006 ± 0.003	4.500	-1.888 (0.163)	0.1091 (0.201)	5.40	0.00	47.07
Paraiba	11	11	1.00 ± 0.06	0.007 ± 0.004	5.145	-6.878* (0.000)	0.0613* (0.000)	5.32	0.00	6656.25
Cabo Frio	14	13	0.99 ± 0.02	0.007 ± 0.004	5.626	-7.041* (0.002)	0.0698* (0.001)	6.30	0.00	4680.00
*Eastern Atlantic*										
Cape Verde	24	15	0.92 ± 0.03	0.003 ± 0.002	2.438	-10.252* (0.000)	0.0523* (0.000)	2.47	0.00	5453.12
Sao Tome	18	10	0.81 ± 0.09	0.002 ± 0.001	1.529	-6.567* (0.000)	0.0714* (0.004)	1.62	0.00	5754.27
Total	183	132	0.99 ± 0.03	0.010 ± 0.007	7.943	-24.555* (0.001)	0.0291* (0.003)	7.43	1.65	32.86

Sample size (N), number of haplotypes (H), haplotype diversity (*h*), nucleotide diversity (π), mean number of pairwise differences within populations (PD), Fu's *Fs *and Ramos-Onsins and Rozas' *R2 *tests followed by their respective *P*-values (in parenthesis), τ (units of mutational time), θ_0 _(function of population size before expansion), θ_1 _(function of population size after expansion) for all populations surveyed. Asterisks (*) indicate significance at a 95% confidence interval.

**Figure 2:**
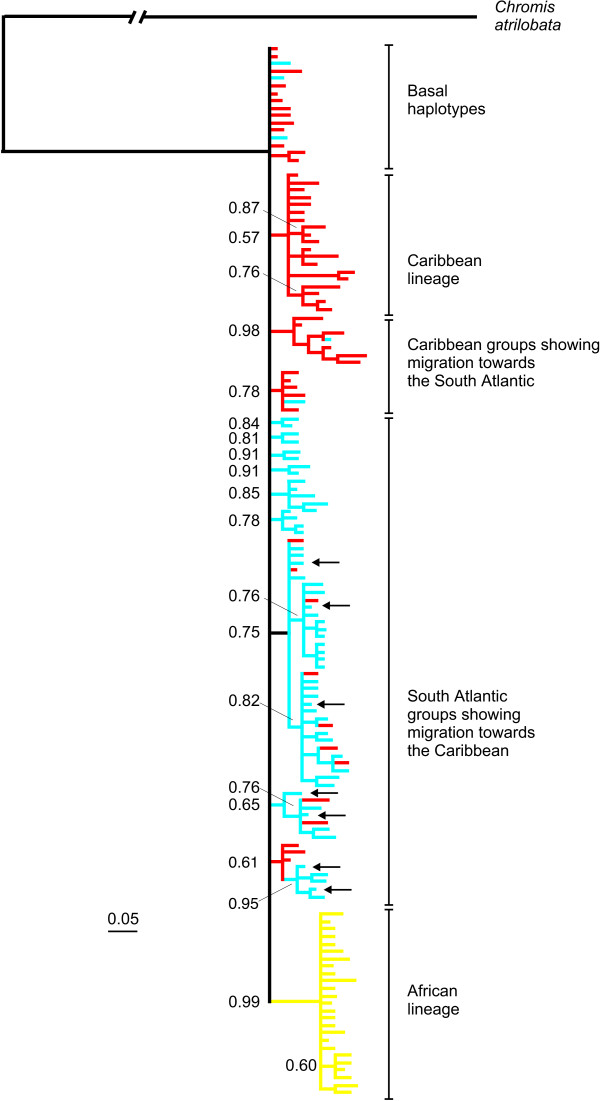
**Phylogenetic tree of *Chromis multilineata *haplotypes**. The 50% majority-rule consensus tree from the Bayesian analysis of *Chromis multilineata *haplotypes. The alternative method of maximum parsimony resulted in almost identical topology. Numbers beside branches correspond to posterior probabilities estimated using the Bayesian approach, and numbers below branches refer to the bootstrap support calculated from the maximum likelihood analysis of 500 sequence replicates assuming model parameters values estimated from Modeltest (see methods). Color code as follows: red, Caribbean; blue, South Atlantic (Brazil + mid-Atlantic Islands); yellow, Africa (Cape Verde + Sao Tome). The branch leading to outgroup was reduced by 50%. Arrows indicate haplotypes shared by the South Atlantic and the Caribbean.

**Figure 3:**
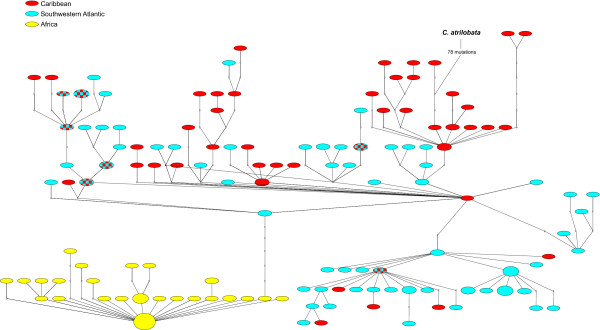
**Parsimony network of *Chromis multilineata *haplotypes**. Statistical parsimony network representing relationships between *Chromis multilineata *haplotypes. Sizes of ovals are proportional to haplotype frequency. Small empty circles represent missing (extinct or unsampled) haplotypes. Colors as in Fig. 2.

Within each of the four biogeographical provinces, pairwise population differentiation (Φ_ST_) values are very close to zero and not significant indicating extensive gene flow among locations (Table 2). Significant population separations (Φ_ST _= 0.033 – 0.21) were observed between Caribbean and South Atlantic (Brazil and Central Atlantic Islands) populations, whereas no significant differences were observed between the Brazilian and Central Atlantic provinces. Interestingly, connections between the southern Caribbean locations (Panama and Grenada) and the South Atlantic were stronger than those between St. Croix (central Caribbean) and the South Atlantic (Table 2). All comparisons between East and West/Central Atlantic populations were significant (Φ_ST _= 0.621 – 0.740), reflecting an evolutionary genetic partition (sequence divergence *d *= 0.88% – 0.98%). AMOVA analysis (Table 3) shows that divergence between the E Atlantic and the remaining provinces accounted for 56.2% of the total molecular variance. Phylogenetic analyses indicate that the ancestral mtDNA lineages are located in the Caribbean (red branches in Fig. 2, red circles in Fig. 3).

**Table 2:** Population pairwise Φ_ST _for *Chromis multilineata*

Locations	1	2	3	4	5	6	7	8	9
1. Grenada	-								
2. St. Croix	0.023	-							
3. Panama	-0.011	0.039	-						
4. Ascension	0.081*	0.173*	0.048	-					
5. St. Helena	0.047*	0.147*	0.009	0.008	-				
6. St. Paul's	0.165*	0.237*	0.108*	0.031	0.065	-			
7. Paraiba	0.019	0.117*	0.005	-0.045	-0.018	0.025	-		
8. Cabo Frio	0.058*	0.166*	0.033*	-0.004	-0.022	0.036	-0.037	-	
9. Cape Verde	0.636*	0.621*	0.621*	0.675*	0.675*	0.692*	0.710*	0.670*	-
10. Sao Tome	0.640*	0.623*	0.624*	0.684*	0.685*	0.710*	0.740*	0.680*	0.004

Asterisks (*) indicate significance at a 99% confidence interval. Significance (*P*) is the probability of finding a variance component and F-statistic that are grater than or equal to the observed values and was tested using a non-parametric approach (Excoffier et al. 1992), with at least 3,000 permutations of the dataset.

**Table 3:** Analysis of molecular variance (AMOVA) of *Chromis multilineata*

Source of variation	Sum of Squares	Percentage of variation	Variance components
Between groups	239.036	56.65	3.575
Within groups	53.533	3.32	0.212
Within populations	439.042	39.99	2.523
Fixation Indices			*P*-value
F_CT_	0.566		0.013
F_SC_	0.077		< 0.001
F_ST_	0.600		< 0.001

Two groups were defined, western Atlantic, comprising the combination of the Brazilian, Caribbean and central Atlantic island populations, and eastern Atlantic, composed of Cape Verde and Sao Tome. Fixation indices are F_CT_: genetic variation between regions; F_SC_: genetic variation among populations within regions; and FST: genetic variation among all populations. Significance (*P*) is the probability of finding a variance component and F-statistic that are greater than or equal to the observed values and was tested using a non-parametric approach (Excoffier et al. 1992), with at least 3,000 permutations of the dataset.

**Figure 4:**
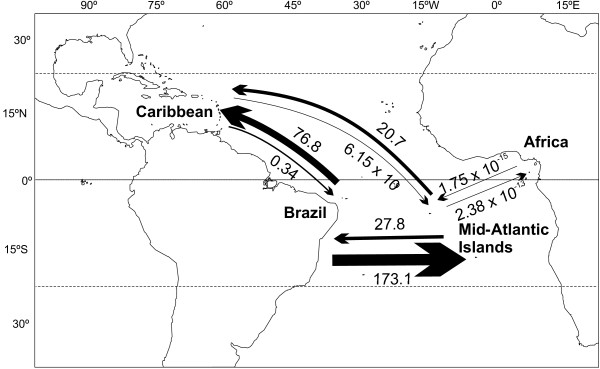
**Migration rates between populations of *Chromis multilineata***. Migration rates between populations of *Chromis multilineata *among the four tropical Atlantic biogeographical provinces, based on the program MIGRATE. Arrows indicate the direction of migration; arrow thickness is proportional to the amount of migration.

**Figure 5:**
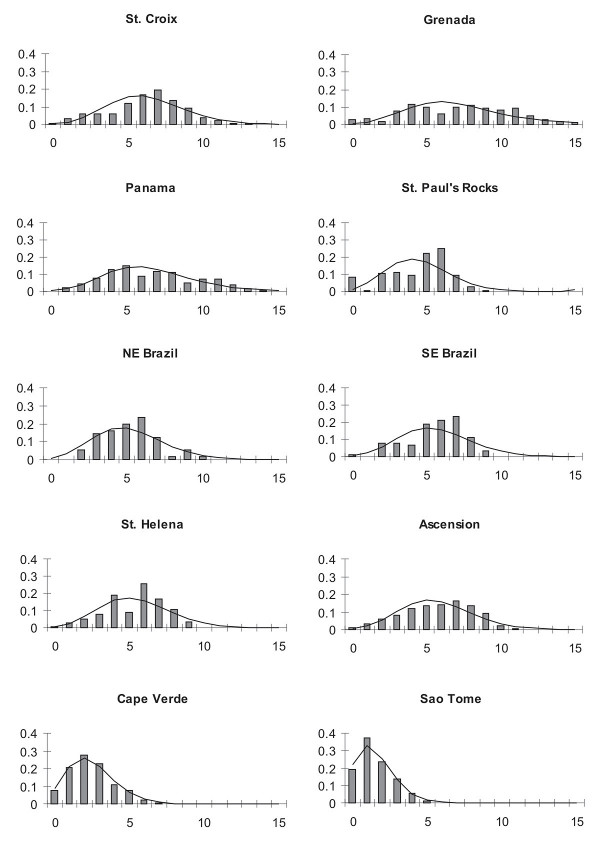
**Mismatch distribution of *Chromis multilineata *haplotypes**. Mismatch distribution of the pairwise number of nucleotide differences among pairs of individuals for the brown chromis (*Chromis multilineata*). Observed distribution represented by vertical bars. Expected distribution as predicted by the sudden population expansion model of Rogers (1995) represented by solid line.

Demographic history was assessed through analysis of pairwise mismatch distributions of all populations (Fig. 4). Caribbean populations have the wider mismatch distributions (13–15 mutations), followed by the Brazilian/Central Atlantic populations (8–10 mutations), then the eastern Atlantic populations (5–7 mutations). Pairwise mismatch distributions (Fig. 5) were not significantly different from values expected under a model of population expansion [39], except for the population at St. Paul's Rocks. Fu's *Fs *and the Ramos-Onsins and Rozas's *R2 *also yield a significant signal for population expansion at all locations except St. Paul's Rocks (Table 1). The coalescence analysis revealed that mutational time (τ) values were similar for western and central Atlantic populations and lower for the eastern Atlantic. Population sizes greater than zero before expansion (θ_0_) were only observed in the Caribbean (Table 1), possibly indicating an older age for this population.

Due to the lack of genetic differences among each of the three Brazilian samples (Paraiba, Cabo Frio and St. Paul's Rocks), among each of the three Caribbean samples (Panama, Grenada and St. Croix), between the two mid-Atlantic locations (Ascension and St. Helena), and between the two eastern Atlantic locations (Cape Verde and Sao Tome), data from each group were combined to calculate bidirectional migration rates among the four provinces. Our analysis shows much migration from both Brazil and the Central Atlantic to the GC, but little in the reverse direction, and virtually none between the western/central Atlantic and eastern Atlantic (the value for this last comparison in Fig. 4 is effectively zero).

**Figure 6:**
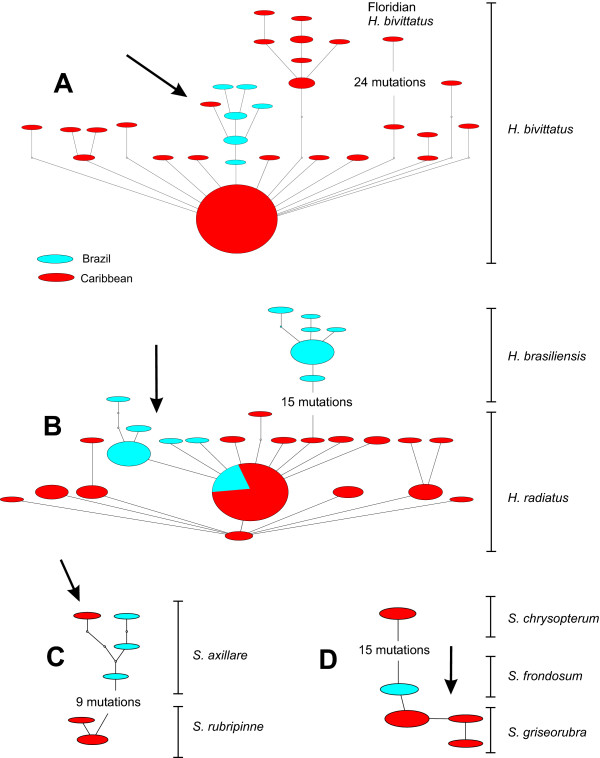
**Parsimony networks of select Atlantic reef fishes**. Statistical parsimony networks representing relationships between: A. cytochrome *b *sequences of *Halichoeres bivittatus *haplotypes; B. cytochrome *b *of *Halichoeres radiatus *and *H. brasiliensis*; C. combined 16s and 12s sequences of *Sparisoma axillare *and *S. rubripinne*; D. combined 16s and 12s sequences of *S. chrysopterum*, *S. frondosum *and *S. griseorubra*. Sizes of ovals are proportional to haplotype frequency. Small empty circles represent missing haplotypes. Arrows indicate points of support for either the center of origin or the center of accumulation hypotheses. Colors as in Fig. 2.

In addition to the *Chromis multilineata *analysis, we built maximum parsimony networks (Fig. 6) using previously published data from the reef fish genera *Halichoeres *[36] and *Sparisoma *[38]. In Fig. 6a, the network of *H. bivittatus *indicates migration from Brazil to the Caribbean. In Fig. 6b, individuals of *H. radiatus *from the Brazilian oceanic islands are derived from the Caribbean group, indicating migration in the reverse direction, with the Caribbean becoming a center of origin. The two *Sparisoma *networks (Fig. 6c and 6d) show that populations and species restricted to the SE corner of the Caribbean are closely related to the Brazilian form.

## Discussion

### Phylogeography of the brown Chromis

Previous genetic surveys of reef organisms in the tropical Atlantic have produced a mosaic of outcomes in terms of the levels of separation among different provinces, with virtually every conceivable pattern being evident [40]. The short-spined sea urchin (genus *Tripneustes*), the red lip blenny (genus *Ophioblennius*), and some wrasses of the genera *Thalassoma *and *Halichoeres *show deep genetic breaks (*d *= 2.0% – 12.7% in mitochondrial DNA coding regions) that correspond to the four major tropical Atlantic biogeographic provinces [20,31,32,41]. In the ocean surgeonfish (*Acanthurus bahianus*), GC populations are separated from those in Brazil and the central Atlantic by an mtDNA sequence divergence of *d *= 2.4%, but no difference is observed between populations at Brazil and the mid-Atlantic islands [34]. Other species show little differentiation throughout the tropical W Atlantic (doctorfish, *A. chirurgus*; pygmy angelfish, genus *Centropyge*; goldspot goby, *Gnatholepis thompsoni *[34,35,42]), or even throughout the entire tropical Atlantic (blackbar soldierfish, *Myripristis jacobus *[37]). In contrast, at the lower end of the spatial scale, some species of wrasses (genus *Halichoeres*) and cleaner gobies (genus *Elacatinus*) show deep genetic breaks and reciprocally monophyletic groups on a scale of tens of km within the GC [36,43,44].

In *C. multilineata*, three levels of genetic diversity were observed: First, the tropical eastern Atlantic populations formed a monophyletic group, separated from all other populations by at least seven diagnostic mutations. Second, there was a significant shift in haplotype frequencies between the GC and the South Atlantic (Brazil + central Atlantic islands) indicating population breaks between the GC and those two provinces (Table 2). Third, there was no detectable genetic difference between the Brazilian and central Atlantic populations. The pattern that emerges from most Atlantic phylogeography studies is that when deep divergences are present, they generally correspond to the major biogeographic provinces, although there are notable instances of strong within-province differentiation (e.g.: gobies and wrasses within both the GC and Brazil [36,43,45]).

In the brown chromis and other reef organisms, the divergence between the eastern Atlantic and the remaining populations (Table 2) may be explained by 1,700 km or more (the shorter straight line distances between the eastern Atlantic and the western/central Atlantic populations are ~1,700 km between St. Paul's Rocks and Cape Verde, ~2,300 km between St. Helena and Sao Tome and ~2,500 km between Ascension and Sao Tome) of unsuitable open-ocean distances, combined with the general east to west direction of prevailing currents at low latitudes. While low but significant pairwise comparisons were observed between the two provinces of the South Atlantic (Brazil and the central Atlantic) and the Caribbean, separated by no more than 2,300 km, no difference was observed between Brazil and the central Atlantic Islands, separated by 2,200 km.

Low genetic structure, such as that seen in *C multilineata *might be expected in organisms with a long pelagic larval stage [34,37] and a similar signal has been observed in the long-spined sea urchin, genus *Diadema *with a ~6 week larval stage [33] and the ocean surgeonfish, *Acanthurus bahianus*, which has a larval duration of ~60 days [34]. *C. multilineata*, however, has a relatively short pelagic larval stage averaging 27 days (33 days maximum [46]), which is substantially less than the average time (48 days based on average current velocity) to cross from Brazil to the mid-Atlantic [47]. However, juvenile *C. multilineata *have been observed in association with floating debris in Belize (L. A. Rocha pers. obs.), and juvenile *Chromis atrilobata *(*C. multilineata*'s sister species in the eastern Pacific) have been observed in open water under floating objects [48,49]; D. R. Robertson pers. obs.). Thus, young *C. multilineata *juveniles survive in the open ocean long after transforming from the larval stage, and relatively strong tropical currents connecting the mid-Atlantic islands and Brazil may transport juvenile *Chromis *between those locations [50,51]. Transport from the western and central Atlantic to the eastern Atlantic evidently has been much less frequent, probably due to larger distances involved in direct transport from W to E Atlantic and smaller source populations for dispersal from the tiny central Atlantic islands (Ascension, St. Helena) to the eastern Atlantic.

The mismatch distribution analysis (Fig. 5), Ramos-Onsins and Rozas's *R*^2 ^test for recent population expansion and Fu's *Fs *neutrality test (Table 1) indicate that all populations have undergone a recent expansion, except for that at the diminutive St. Paul's Rocks. This expansion was probably caused by the ~10 fold increase in suitable shallow reef habitat associated with the sea-level rise of ~130 m since the last glacial maximum [52]. St. Paul's Rocks is an exception: because there is no shelf at that island, which is a pillar rising vertically from deep water [29], available habitat probably hasn't increased with sea-level rise. Thus the lack of an expansion signal may reflect a relatively constant population size due to stability in habitat availability. The lower number of pairwise differences, and haplotype and nucleotide diversities observed at Cape Verde and Sao Tome in the eastern Atlantic, indicate a more recent expansion at those locations than in the western and central Atlantic. In addition to lower sea-levels and corresponding reduced habitat available during the last glacial period in the eastern Atlantic, water temperatures in that area were lowered more than in the western and central Atlantic by enhanced coastal upwelling [53], a stress on tropical species that may have caused strong fluctuations or reductions in population size.

Migration patterns among populations (Fig. 4) are consistent with the general direction of surface current flows (Fig. 1): the highest rates are from the South Atlantic to the Caribbean (coinciding with the flow of the North Brazil current) and from Brazil to the mid-Atlantic islands (coinciding with the flow of the South Atlantic Gyre). In accordance with the phylogenetic results, migration between western and eastern Atlantic (a direction that opposes major surface currents; Fig. 1) was effectively zero.

### The Greater Caribbean hotspot: center of origin and center of accumulation

Our phylogeographic analysis of *C. multilineata *provides two lines of evidence relevant to the mechanism(s) that produced the GC hotspot of diversity. First, supporting accumulation, the short (i.e. relatively young) branches are mostly in Brazil and the central Atlantic islands (in Fig. 2 ~75% of the short branches are blue, endemic to Brazil and the two mid-Atlantic islands). However, the short branches also include a few Caribbean haplotypes (~25% of short branches in the mostly South Atlantic lineage in Fig. 2), indicating that the lineages diversified in the South Atlantic, and that individuals carrying those haplotypes recently arrived in the Caribbean. This South to North pattern of dispersal is consistent with the pattern of oceanic current flow (Fig. 1) and migration rates (Fig. 4), and indicates that the hotspot of diversity in the Caribbean has acted recently as a CA.

Similarly to what we describe here for *C. multilineata*, the Panamanian sample of the wrasse *Halichoeres bivittatus *has one individual (among 23 sampled) with a haplotype that is more similar to those found in Brazil than those in other Caribbean locations (Fig. 6a), indicating the recent arrival of a haplotype of South Atlantic origin [36]. Additionally, in the goby genus *Gnatholepis*, and the angelfish genus *Centropyge*, the Atlantic species are recently derived from much more diverse Indo-Pacific groups, and apparently only recently arrived in the GC from the Indian Ocean via the South Atlantic [35,42,54], supporting accumulation of species at the GC hotspot.

The phylogeny of parrotfishes (genus *Sparisoma*) also supports accumulation at the Caribbean (Fig. 6c and 6d). The parrotfish *S. axillare *is abundant and widely distributed throughout the Brazilian coast, but known only from SE Venezuela in the Caribbean, indicating that the population there is probably a result of recent dispersal from Brazil. Likewise, the parrotfish *S. griseorubra *is restricted to the southern Caribbean (SE Venezuela) and its sister species is *S. frondosum *(endemic to Brazil), indicating that *S. griseorubra *likely originated from ancient dispersal by the ancestor of the *S. frondosum/S. griseorubra *lineage. The splitting of *S. frondosum *and *S. griseorubra *alternatively could be explained by sympatric speciation in the Caribbean (the groups with Brazilian affinities co-occur with their Caribbean counterparts in the southern Caribbean) followed by dispersal towards Brazil. However, speciation with gene flow in reef fishes has only been inferred when there are strong ecological gradients [36], or where the fish are strictly associated with coral hosts [55]. As this is not the case for *Sparisoma*, this alternative is less likely than an allopatric split between Brazil and the Caribbean after dispersal northwards by a Brazilian lineage.

Supporting origin at the Caribbean, almost all basal haplotypes of the *C. multilineata *tree are observed only in Caribbean individuals (Figs. 2 and 3), which indicates that populations at the three other regions are derived from a Caribbean ancestor. Moreover, the Caribbean samples (either separately or in combination) have the widest mismatch distributions (spanning 13 to 15 mutations; Fig. 5) indicating that this region hosts the oldest and most stable population. We can also infer that, because all eastern Atlantic haplotypes apparently have a single origin (monophyly in Fig. 2), they most likely originated from a single colonization event (Fig. 3) on the order of two hundred thousand years ago (based on our trans-isthmian molecular clock estimate of 4.4%/Myr). The recent finding of *Centropyge aurantonotus *in the eastern Atlantic (previously known only from the western Atlantic, but recently observed in low numbers in Sao Tome [56]) and the general direction of trans-Atlantic range expansion in the gold spot goby [35] support this rare west to east colonization route.

Lending further support to the CO hypothesis, populations of the wrasse *H. radiatus *in Brazilian oceanic islands are much more closely related to Caribbean *H. radiatus *than to populations of *H. brasiliensis *(its sister species) in the adjacent coast line, indicating that these islands were colonized by migrants of Caribbean origin (Fig. 6b). A similar pattern of southward dispersal from the GC to the offshore Brazilian islands has been observed in gobies of the genus *Bathygobius *[45]. Colonization outward from the Caribbean must be a rare event because it goes against prevailing currents, but as our analysis indicates, it has happened and can lead to the establishment of new populations, supporting the CO hypothesis.

Support for origin of diversity in the GC comes from other recent phylogenetic and biogeographic analyses. A survey of seven-spined gobies [57] shows that two peripheral species of *Elacatinus *(*E. figaro *from Brazil and *E. puncticulatus *from the eastern Pacific) are older species whereas the 10 newest (youngest) *Elacatinus *species are found only in the Caribbean biodiversity hotspot. That is, recent speciation that produced 83% of the species in this genus occurred within the GC. Likewise, the diverse families Chaenopsidae and Labrisomidae are represented in the GC by 45 species each, but elsewhere in the Atlantic there are only four and 11 Atlantic species in each of these families respectively [40,58,59]. Hamlets (genus *Hypoplectrus*) include as many as a dozen or more closely related "species" and are restricted entirely to the GC [60]. Thus, speciation leading to considerable faunal enrichment most likely occurred *in situ *within the GC in those four taxa.

Recent phylogenetic analyses of large reef fish groups also provide useful information to this debate. Among wrasses (Labridae), the Caribbean and Eastern Pacific *Halichoeres *are for the most part monophyletic, indicating that they diversified *in situ *and supporting CO in a recent time scale, but at the same time their diverse group of ancestors is composed of Indo-Pacific species [21,61] supporting CA deeper in time, on a scale of tens of millions of years. Similarly to the wrasses, the Caribbean groupers of the genera *Epinephelus *and *Mycteroperca *are monophyletic groups derived from Indo-Pacific ancestors [62], supporting both ancient CA and more recent CO. The Caribbean butterflyfishes are mostly a paraphyletic assemblage of lineages derived from more diverse Indo-Pacific groups, supporting CA [63]. Within the damselfishes, the Caribbean (and Atlantic) species *Abudefduf saxatilis *seems to be a recent arrival from the Indo-Pacific (it is very closely related to a group containing eight Indo-Pacific and one eastern Pacific species), also supporting CA [64]. Even though these large scale phylogenies are useful, most lack peripheral (mostly Brazilian) endemics, making their contribution to this debate limited. However they provide an excellent framework that, with the addition of a few key species, may become an important piece in the tropical biodiversity puzzle.

## Conclusion

Our data indicates that the Greater Caribbean is both a center of origin and accumulation for genetic lineages within species (e.g. *Chromis multilineata *and *Halichoeres bivittatus*) and for sister species within genera (e.g. *Sparisoma*). Such bidirectional dispersal is also reflected in the geographic distributions of tropical Atlantic fishes and invertebrates: several species that are widely distributed in the Brazilian coast are also recorded in the southeastern corner of the Caribbean [30,65,66]. Likewise some widely distributed Caribbean species also occur in northern Brazil, sometimes on only a few reefs south of the Amazon outflow, evidence of recent southward dispersal [30,40].

We conclude, on the basis of multiple lines of evidence, that the GC marine biodiversity hotspot did not arise through the action of a single mode of microevolutionary change. This diversity is the product of a more complex and idiosyncratic process in different taxa, and it is clear from the accumulating data that several mechanisms have contributed. The hypotheses of center of origin and center of accumulation are not mutually exclusive, and acting in concert, as they have done in the GC, origin and accumulation can generate more diversity than either process acting alone.

In closing we note that the IMA is a much larger hotspot than the GC, in terms of both geography and biodiversity. The IMA hotspot is flanked on both sides by the numerous archipelagos of the Pacific and Indian Oceans, a feature nearly absent for the GC hotspot. Moreover, the GC is marked by a turbulent history involving extinctions of many reef-associated organisms in the past few million years [67], a feature not yet detected at the IMA. We suggest that the larger size and greater stability of the IMA, combined with its extensive halo of peripheral habitats, serves to strengthen the *biodiversity feedback *between hotspots and other areas, and contribute to the global center biodiversity in the IMA. Finally, it remains to be seen whether the principles of origin and accumulation apply to terrestrial biodiversity, or whether these evolutionary mechanisms are restricted to high-dispersal media, the exclusive domain of the world's oceans.

## Methods

### Sampling strategy

*Chromis multilineata *is a common reef fish that is widely distributed on both sides of the tropical Atlantic, as well as at the mid-Atlantic islands [68]. They have demersal eggs that develop into pelagic larvae in about three days [69,70]. Estimates of pelagic larval duration range from 24 to 33 days [46]. Adults can be locally very abundant and are usually found in schools from a few to several hundred individuals, swimming and feeding on plankton above the reef [71].

A total of 183 specimens of *Chromis multilineata *were obtained from 10 locations, which included at least two locations within each of the four provinces (Table 1, Fig. 1). Specimens were collected with polespears while scuba diving or snorkeling, between 1997 and 2002. Tissues (muscle and/or gill) were stored in a saturated salt-DMSO buffer (Amos and Hoelzel 1991). A recent phylogenetic survey of damselfishes indicates that the eastern Pacific species *C. atrilobata *is the sister of *C. multilineata *[64]. To confirm this relationship we sequenced *C. atrilobata *from Cocos Island (*n *= 3) and Panama (*n *= 3) and other species of the genus in the Atlantic that were not included in the phylogeny [64]: *C. scotti *from Florida (n = 2); *C. enchrysura *from Florida (n = 2), *C. limbata *from the Azores (*n *= 4) and *C. lubbocki *from Cape Verde (*n *= 3). The resulting tree (not shown) supports the conclusion that *C. multilineata *and *C. atrilobata *are sister species.

### DNA Extraction and Sequencing

Total genomic DNA was extracted using QIAGEN (Valencia CA) Dneasy extraction kits following the manufacturer's protocol. Extracted DNA was frozen in TE buffer and archived at -20°C. Primer names indicate the DNA strand (H = heavy and L = light strand) and the position of the 3' end of the oligonucleotide primer relative to the human mitochondrial DNA sequence. A segment of 802 base pairs of the mtDNA cytochrome *b *gene was amplified with the primers L14725 (5' GTG ACT TGA AAA ACC ACC GTT G 3') and H15573 (5' AAT AGG AAG TAT CAT TCG GGT TTG ATG 3') [72].

Thermal cycling in polymerase chain reactions (PCR) consisted of an initial denaturing step at 94°C for 1 min 20 sec, then 35 cycles of amplification (40 sec of denaturation at 94°C, 30 seconds of annealing at 52°C, and 55 sec of extension at 72°C), and a final extension of 2 min 30 sec at 72°C. Each tube contained 15 μl of water, 2.5 μl of MgCl2, Mg free buffer and DNTPs, 0.3 μl of each primer, 0.4 μl of Taq DNA polymerase and 1.5 μl of purified DNA. Excess primers were removed through simultaneous incubation of PCR product with exonuclease I and shrimp alkaline phosphatase (USB Corp., Cleveland OH).

Sequencing reactions with fluorescently-labeled dideoxy terminators (BigDye) were performed according to manufacturer's recommendations, and analyzed with an ABI 377 automated sequencer (Applied Biosystems, Inc. Foster City, CA). All samples were sequenced in the forward direction (with L14725 primer), but rare and questionable haplotypes were sequenced in both directions to ensure accuracy of nucleotide designations. Representative haplotype sequences are deposited in GenBank under accession numbers EU431997 – EU432046. Copies of the complete data set are available from LAR.

### Phylogenetic and Population Analyses

Sequences were aligned and edited with Sequencher version 3.0 (Gene Codes Corp., Ann Arbor, MI). Population structure and gene flow were assessed with an analysis of molecular variance AMOVA [73] in the program Arlequin version 3.0, which generated Φ_ST _values (a molecular analog of F_ST _that includes sequence divergence among haplotypes as well as haplotype frequency shifts; [74]). Genetic variation is described with nucleotide diversity (*π*; equation 10.19 [75]) and haplotype diversity (*h*; equation 8.5 [75]) within each location.

The computer program MODELTEST version 3.06 [76] was used to determine the substitution model that best fits the data through a minimal theoretical information criterion (AIC). The model chosen was TRN+Γ [77] with a gamma distribution of 0.97 to estimate sequence divergences (*d *values) between haplotypes. Equal weighting of all three codon positions was used. Relationships between all haplotypes and closely related species were estimated using a Bayesian phylogenetic analysis performed with MrBayes 3.1 [78]. Preliminary runs were performed to monitor the fluctuating value of the likelihoods of the Bayesian trees, and all parameters appear to reach stability before 250,000 generations. The Markov chain analysis was run for 20 million generations. A burn-in period, in which the initial 10,000 trees were discarded, was adopted and the remaining tree samples were used to generate a 50% majority rule consensus tree. The posterior probability of each clade was then provided by the percentage of trees identifying the clade [79]. In addition, the software PAUP 4.0b10 [80] was used to conduct maximum parsimony (MP) and neighbor-joining analyses that were evaluated with 1000 bootstrap replicates implemented with PAUP* version 4.0b10 [80]. The minimum evolution criterion was used by applying maximum likelihood distances estimated with the model chosen by Modeltest in the neighbor-joining analysis. The resulting Bayesian, parsimony and neighbour joining trees were not significantly different.

Departure from neutrality was tested using Fu's *Fs *[81] and Ramos-Onsins and Rozas' *R2 *statistic [82]. The *R2 *measure is based on the difference between the number of singleton mutations and the average number of nucleotide differences among sequences within a population sample. Both *Fs *and *R2 *are powerful tests used to detect recent population expansions under assumptions of neutrality [81,82]. Significance of *R2 *and *Fs *were evaluated by comparing the observed value with a null distribution generated by 10,000 replicates, using the empirical population sample size and observed number of segregating sites implemented by DnaSP version 4.10.9 [83]. Moreover, in order to estimate the demographic parameters of past population expansions we calculated mismatch distributions (or the distribution of the pairwise genetic distances) for all populations using DnaSP. Time and magnitude of the inferred population expansion were determined by calculating θ_0_, θ_1 _and τ, where θ_0 _= 2N_0μ _(N_0 _= population size before expansion); θ_1 _= 2N_1μ _(N_1 _= population size after expansion); and τ = 2μ*t *(μ = mutation rate per site per generation; *t *= time in generations).

The mutation rate per lineage per year (λ) was estimated by solving the formula λ = *d*/2*T*, where *d *is the genetic distance between *C. multilineata *and *C. atrilobata*, and *T *is the time since divergence between the two species used. We used a *T *of 3.5 million years (Myr), as that is the upper limit age for the final closure of the Isthmus of Panama [84]. The rate of 2.21%/Myr (within lineages) that we estimated is very similar to that obtained in a recent survey of *Chromis *using a slightly different portion of the cyt *b *gene (2.36%/Myr; [85]. The closure of the Isthmus of Panama was used in several other studies to estimate mutation rates in reef fishes (e.g.: [35,86-88]).

Migration rates between the major biogeographical provinces (Brazil, the Caribbean, central Atlantic islands and eastern Atlantic islands) *Nm *(where *N *is effective female population size and *m *is migration rate) were calculated with the software MIGRATE version 1.7.6 [89], which uses a maximum likelihood approach based on coalescence theory [90]. Estimators of migration rates based on coalescence theory can detect asymmetries (directionality) in migration rates and differences in population sizes, a considerable advantage when testing migration rates in the context of evolutionary theory [91]. Most of the default settings for MIGRATE were used on the first run, but the number of trees sampled was increased to 10,000 for the short chains and 100,000 for the long chains to avoid local maxima on the likelihood surface. A trial run was used to generate the input parameters; the average of results of 10 runs (number of trees sampled was progressively increased from 10,000 to 300,000 for the short chains and from 100,000 to 3,000,000 for the long chains) is reported here. We also used the software IM to estimate migration between populations of *C. multilineata*; IM uses a Markov chain Monte Carlo approach [92]. The results from the IM analysis are not shown because they were completely compatible with the results from MIGRATE.

In addition, we re-analyzed data from four groups of reef fish species (the wrasse genus *Halichoeres *[36], and the parrotfish genus *Sparisoma *[38]) to specifically assess predictions from the center of origin and center of accumulation hypotheses. To do so we constructed statistical parsimony networks for those species as well as *Chromis multilineata *using the computer program TCS version 1.21 [93] and analyzed the geographical distribution of mtDNA haplotypes.

## Authors' contributions

LAR obtained samples from the western Atlantic, carried out the molecular genetic analyses and prepared the manuscript. CRR participated in DNA sequencing and population genetic analysis. DRR collected samples at the Central Atlantic Islands, the Caribbean, the Eastern Pacific and Africa, and helped prepare the manuscript. BWB participated in the design and coordination of the study and helped prepare the manuscript. All authors edited and approved the final manuscript.
